# Estimation of sequencing error rates in short reads

**DOI:** 10.1186/1471-2105-13-185

**Published:** 2012-07-30

**Authors:** Xin Victoria, Natalie Blades, Jie Ding, Razvan Sultana, Giovanni Parmigiani

**Affiliations:** 1 Department of Biostatistics and Computational Biology, Dana-Farber Cancer Institute, Boston, MA 02215, USA; 2 Department of Biostatistics, Harvard School of Public Health, Boston, MA 02115, USA; 3 Department of Statistics, Brigham Young University, Provo, UT 84602, USA; 4 Department of Bioinformatics, Boston University, Boston, MA 02215, USA

## Abstract

**Background:**

Short-read data from next-generation sequencing technologies are now being generated across a range of research projects. The fidelity of this data can be affected by several factors and it is important to have simple and reliable approaches for monitoring it at the level of individual experiments.

**Results:**

We developed a fast, scalable and accurate approach to estimating error rates in short reads, which has the added advantage of not requiring a reference genome. We build on the fundamental observation that there is a linear relationship between the copy number for a given read and the number of erroneous reads that differ from the read of interest by one or two bases. The slope of this relationship can be transformed to give an estimate of the error rate, both by read and by position. We present simulation studies as well as analyses of real data sets illustrating the precision and accuracy of this method, and we show that it is more accurate than alternatives that count the difference between the sample of interest and a reference genome. We show how this methodology led to the detection of mutations in the genome of the PhiX strain used for calibration of Illumina data. The proposed method is implemented in an R package, which can be downloaded from http://bcb.dfci.harvard.edu/∼vwang/shadowRegression.html.

**Conclusions:**

The proposed method can be used to monitor the quality of sequencing pipelines at the level of individual experiments without the use of reference genomes. Furthermore, having an estimate of the error rates gives one the opportunity to improve analyses and inferences in many applications of next-generation sequencing data.

## Background

The rapid development of new DNA sequencing technologies is transforming biology by allowing individual investigators to sequence volumes previously requiring a major genome center. Before the development of next-generation sequencing platforms, Sanger biochemistry was the basis of sequencing production. Sanger sequencing, or conventional sequencing has been fine-tuned to achieve read-lengths of up to ∼1,000 bp and per-base accuracies as high as 99.999% [1]. However, given several bottlenecks in conventional sequencing that restrict its parallelism, the optimization in throughput and cost has reached a plateau. Several alternative sequencing strategies have been proposed in the past several years to reduce sequencing time and cost.

One category of such alternatives is cyclic-array sequencing, referred to as second-generation or next-generation sequencing, which has been made available commercially, and includes products such as the 454 Genome Sequencers (Roche Applied Science), the Illumina (Solexa) platform (Illumina), the SOLiD platform (Applied Biosystems) and the HeliScope Single Molecule Sequencer (Helicos). Because of the much higher degree of parallelism and much smaller reaction volumes, next-generation sequencing achieves much higher throughput with dramatically lower cost. The disadvantages are shorter reads and higher error rates compared to Sanger sequencing. Next-generation sequencing has been applied in many areas of biology, including quantification of gene expression and alternative splicing, polymorphism and mutation discovery, microRNA profiling, and genome-wide mapping of protein-DNA interactions. A detailed review of sequencing technologies can be found in [1].

Aware of the large impact of sequencing quality on downstream analysis, several groups have attempted to detect, quantify and understand errors that arise from next-generation sequencing pipelines. At the nucleotide level, base-calling algorithms developed by manufacturers (for example, Bustard by Illumina) and independent investigators [2-5] provide per-base phred-like [6] quality scores as a byproduct. These methods require fluorescence intensity measurements from sequencing runs as input, and the per-base quality scores produced need to be further summarized to assess sequencing quality at higher levels. At the technology level, there have been efforts to characterize error patterns associated with different platforms, which include Dohm *et al*. and Hansen *et al*. [7,8] for the Illumina platform, and Huse *et al*. [9] for the 454 Genome Sequencers. These studies are very important in facilitating our understanding of the quality characteristics, however, they do not provide methods to assess the quality of sequencing data that are produced everyday in individual laboratories.

Sequencing quality also needs to be evaluated and analyzed in light of different applications. For example, Bullard *et al*. [10] conducted a study of statistical methods for normalization and differential expression (DE) analysis of Illumina transcriptome sequencing data. They evaluated how DE results are affected by varying gene lengths, base-calling calibration methods, and library preparation effects. They obtained the number of uniquely mapped reads with 0 (U0), 1 (U1) or 2 (U2) mismatches and used the ratio (U1+U2)/(U0+U1+U2) to estimate the per-read sequencing error rate, which is the proportion of reads that contain at least one error. We refer to this method of counting the number of mismatches to the reference genome as the *mismatch counting* method. This builds on the assumption that the perfect-matching reads contain no errors and that U1 and U2 contain sequencing errors but not SNPs. It also requires mapping to a reference genome, a step that may be problematic in applications such as threat detection, as the genome sequence of interest may not be known.

Tools also exist to correct errors from next generation sequencers [11-17] and error rates can be estimated as a by-product. These methods are based on *k*-mer or substring frequencies, or finding overlaps between reads, which are very computationally intensive, require a large amount of memory, and are difficult to work with large genomes.

In this work we develop a simple and efficient method to estimate the error rates in any sequencing pipelines based on the observation that there is a linear relationship between the number of reads sequenced and the number of reads containing errors. We refer to this proposed method as *shadow regression*, and show that it works well in applications with moderate to high sequencing depth, such as mRNA-seq, re-sequencing and SAGE.

As with any high throughput experiments, it is important to monitor the quality of next-generation sequencing data at the level of individual experiments. Currently the percentage of reads mapped is used as a quality indicator but it does not directly address the fundamental question of how much error is present in the reads obtained from a sample. A reference genome may not be available at all. Even if the reference genome is available, we show, using simulated data and real data on PhiX, that mapping reads to the reference genome can introduce biases even at relatively modest polymorphism rates. Furthermore, having an estimate of the error rates gives one the opportunity to improve analyses and inferences in many applications of next-generation sequencing data. For example, error rates are useful in understanding the fidelity of SNP or mutation calls as a function of coverage.

## Methods

### Next-generation sequencing and SAGE data

Public next-generation sequencing data were obtained from the NCBI Sequence Read Archive (SRA) [18]. The SRA accession numbers are: SRA010153 (mRNA-seq, MAQC), SRA001150 (mRNA-seq, Encode) and SRA010105 (re-sequencing, mutation screening). In addition, PhiX data were generated on Illumina Genome Analyzer II by the Center for Cancer Computational Biology at Dana-Farber Cancer Institute (DFCI). SAGE data were obtained from NCBI SAGEmap [19]. Next-generation sequencing data were downloaded in FASTQ format and converted to read counts. SAGE data were downloaded as read counts. Reads that contain N’s (no calls) or consist of all A’s, C’s, G’s or T’s are filtered out before shadow counts are computed. More details about the data can be found in the Results and Discussion section.

### Estimating sequencing error rates

**Figure 1:**
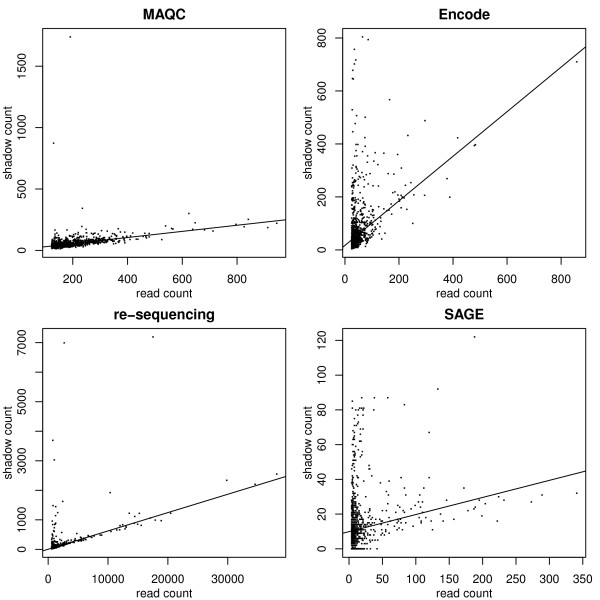
**Some examples of read-shadow relationships from different data sets.** The solid lines are robust regression lines fit to the data.

**Figure 2:**
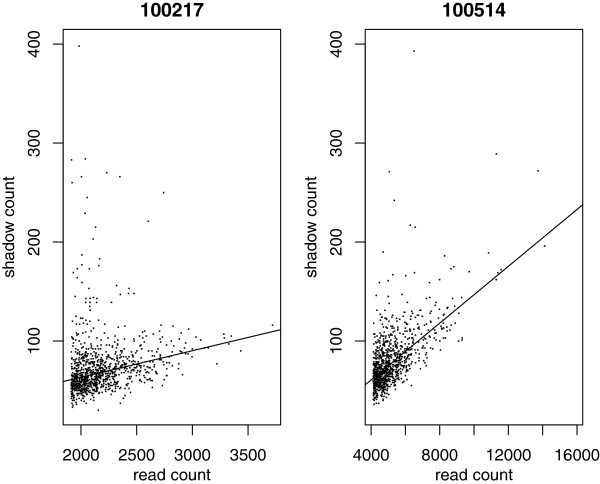
**Two PhiX samples run on Illumina Genome Analyzer II: scatter plots of read count and shadow count.** The solid lines are robust regression lines fit to the data.

Sequencing pipelines output many short sequence reads representative of the sequences in the sample. If substitutions, insertions or deletions occur, the resulting sequence read differs from the true sequence. Given a read *t*, we refer to reads that have sufficient sequence similarity with *t* as the shadows of *t*. The results shown here are based on differences by up to two bases, though different definitions could be adopted depending on the application. Among the shadows, there may be sequences that are legitimate and error-free. Our method is based on the observation that the number of shadows due to sequencing errors increases linearly with the read count of *t*, while the number of legitimate shadows is independent of the read count of *t*. Figure 1 shows read-shadow relationships for samples from some of the data sets to which shadow regression was applied. The same plots for two DNA sequencing runs of the bacteriophage PhiX are shown in Figure 2. We see that as read count increases, shadow count also increases. Infrequently occurring reads have wildly varying numbers of shadows; however, among frequently occurring reads, the relationship between read count and shadow count is clear and positive. We propose a linear model based on this observation to estimate error rates in sequencing pipelines, which we call *shadow regression*. Even though there may be more than two errors in a read, as is the case for reads containing sequence-specific errors shown in Nakamura *et al*. [20], we found that using reads that differ from *t* by up to two bases in shadow regression gives us accurate estimates of error rates without excessive computational cost. Note that we present results with substitution errors only in this paper; application of this method to insertions and deletions differ only in the way shadows are defined and are implemented in the R package. Insertions at the beginning of reads and deletions at the end of reads may result in genuine reads that are shifted by one base. Therefore the resulting estimates of position-specific indel rates may be biased, so these estimates are not computed at the extremes. Read-level indel rates are unlikely to be affected.

#### Per-read error rates

Let *r*_*t *_denote the true number of reads with sequence *t* in a given sample, and

(1) $$r_{t}=n_{t}+e_{t},$$

where *n*_*t *_is the number of reads with sequence *t* that have been sequenced correctly and *e*_*t*_ is the number of reads that come from sequence *t*, but contain sequencing errors and are therefore slightly different from *t*. We define the per-read error rate to be the proportion of reads containing sequencing errors among all the reads in a sample, which is the same as the proportion of reads containing errors per additional unit of reads sequenced:

(2) $${\text{Error Rate}}_{\text{read}}=\frac{\underset{t}{\sum }e_{t}}{\underset{t}{\sum }r_{t}}=\frac{\Delta e_{t}}{\Delta r_{t}}.$$

Based on our observation of the linear relationship between the number of shadow reads due to sequencing errors and the number of true reads for a given sequence *t*, we propose the following linear model to estimate sample-specific error rates in sequencing runs. In the case of Illumina Genome Analyzer (Solexa), a sample corresponds to a lane in a flow-cell. Our model is

(3) $$s_{t}=\alpha +\beta n_{t}+\varepsilon ,$$

where *s*_*t*_ denotes the number of observed shadows of sequence *t* (defined to be reads that differ from sequence *t* by up to two bases) in the sample, *ε* is independent of *n*_*t*_ and approximately Gaussian with mean 0 and variance *σ*^2^, *α* is the intercept, and *β *is the parameter that represents the slope. Because shadows can also come from legitimate reads that are error free, *β* needs to be estimated robustly so that they are not influenced by error-free shadows. Shadow counts are computed by first enumerating all possible shadows of each observed read, and then identifying the shadows that are actually observed.

It is generally observed that in a given sample, a small number of reads have high frequencies while the majority of reads have very low frequencies. We find that the relationship between the number of reads and the number of shadows is captured in high frequency reads, and that it is enough to use the top 1000 reads with highest frequencies to estimate *β*. We also regard the top 1000 reads to be error free, and thus exclude them from shadow counts of any read.

The estimated slope $\hat{\beta }$ is the number of additional error shadows we get for each additional unit of correctly sequenced read *t*, *i.e.*$\hat{\beta }=\Delta e_{t}/\Delta n_{t}$. The per-read error rate we want to estimate can be obtained the following way:

(4) $${\text{Error Rate}}_{\text{read}}=\frac{\Delta e_{t}}{\Delta r_{t}}=\frac{\Delta e_{t}}{\Delta n_{t}+\Delta e_{t}}=\frac{\hat{\beta }}{1+\hat{\beta }}.$$

Thus, by examining the slope, we may obtain a sample-specific estimate of the sequencing error rate. The error due to substitutions, insertions or deletions may be estimated separately with this method. Alternatively, the aggregate error from any source may be estimated.

#### Position-dependent error rates

It has been observed that error rates in sequencing pipelines depend on the base position in the read [5,7], which can be estimated by stratifying shadow reads by position. We define the per-base error rate at position *i* to be the proportion of reads with sequencing errors at position *i* among all the reads in the sample, *i.e.*,

(5) $${\text{Error Rate}}_{\text{base}}^{i}=\frac{\underset{t}{\sum }e_{t}^{i}}{\underset{t}{\sum }r_{t}}=\frac{\Delta e_{t}^{i}}{\Delta r_{t}^{i}},$$

where $e_{t}^{i}$ is the number of reads that should be *t* if error free, but are not because of at least a sequencing error at position *i*. The corresponding linear model is

(6) $$s_{t}^{i}=\alpha_{i}+\beta_{i}n_{t}+\varepsilon^{i},$$

where $s_{t}^{i}$ is the number of error shadows that differ from read *t* at least at position *i*, and *ε*^*i*^ is Gaussian with mean 0 and variance *σ*^*i*2^. As with per-read error rates, ${\hat{\beta }}_{i}/(1+{\hat{\beta }}_{i})$ is the per-base error rate at position *i* that we want to estimate.

### Application to different data types

Our method, shadow regression can be applied to mRNA-seq, SAGE, re-sequencing and whole genome sequencing data regardless of platform, as long as there is sufficient coverage in the input data. The current implementation requires the read lengths from a given sample to be the same. Precautions need to be taken when working with whole genome sequencing data as some genomes are highly repetitive and may result in over estimation of the error rates. One way to assess whether a particular genome is too repetitive to apply shadow regression directly is to sample reads from the reference genome and see if shadow regression gives an error rate estimate that is very close to 0. If this error rate is significantly greater than 0, indicating a genome with repetitive regions, one can mask out reads from the repetitive regions or retain only reads from the coding regions before applying shadow regression. The test for repetitive genomes described here is implemented in the R package.

### Required coverage

Shadow regression requires sufficient coverage to estimate the error rate accurately. For mRNA-seq, samples shown in the results section for MAQC and Encode human data contain about 12 million reads, which is sufficient for shadow regression to work well. When using about 1.2 million reads sampled from the 12 million reads as input, shadow regression still gives good estimates (data not shown). Therefore we are confident that our method works well with any mRNA-seq data. In general, we would like the top 1000 read counts, which is what we use for error estimation, to have a range of about 500 or more. Therefore the maximum read count needs to reach about 500 or more.

## Results and discussion

### Simulation studies

In order to determine the accuracy of our proposed method, we performed two simulation studies. The first one assumes that errors in a read occur independently of each other, while the second study assumes that once an error occurs in a read, it is more likely to make another error in that read. For both studies, we start with U0 reads (reads that are uniquely mapped to the reference genome with no mismatches) of an MAQC experiment 2 sample (SRR037440). These reads, which map to the reference genome perfectly, are assumed to be the error-free ones for the purpose of generating synthetic data. Substitution errors are then added to these reads according to pre-specified position-specific error rates. For the second study where the errors do not occur independently of each other, the error rates for the rest of the read double once an error occurs in a read, *i.e.* if an error occurs at position *i*, the error rates for positions *j *>* i* become twice their pre-specified error rates. The pre-specified error rates are based on the estimated error rates of sample SRR037440 by counting the number of mismatches to the reference genome at each position. This set of position-specific error rates are then scaled to create a range of different error rates. The estimated error rates were calculated by transforming the slope from a robust linear regression (as implemented in the *rlm()* function in the R library MASS). For each set of pre-specified error rates, we repeated the error simulation and estimation one hundred times.

**Figure 3:**
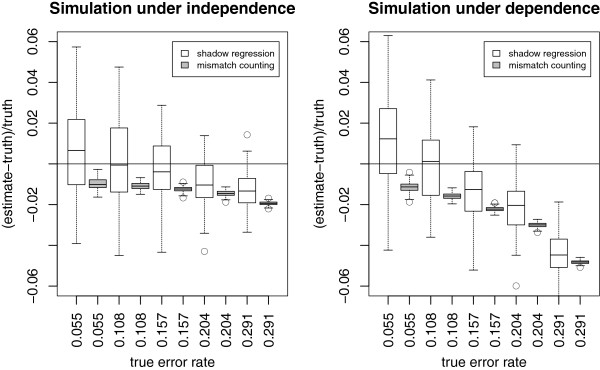
**Simulations: per-read error rate estimates based on simulated reads under five different error rates.** The horizontal lines are drawn at 0, where the error rate is estimated perfectly. Each boxplot contains the differences between error rate estimates and the true error rate from 100 simulated data sets. Shadow regression and mismatch counting estimates are plotted next to each other for a given error rate.

**Figure 4:**
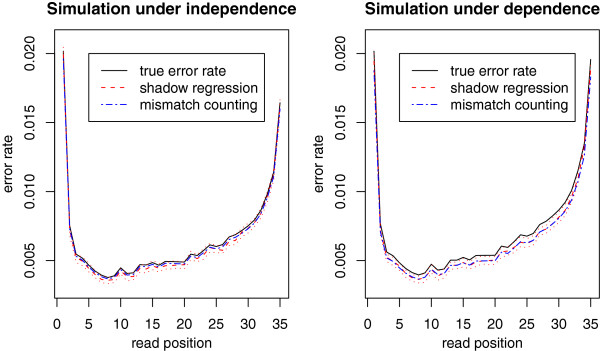
**Simulations: position-specific error rate estimates for simulated data with corresponding per-read error rate of 0.20.** The red dotted lines are the upper and lower quartiles of the shadow regression estimates (N=100).

Figure 3 shows the performance of shadow regression for estimating per-read error rates. Under the independent error model, shadow regression gives estimates that are usually within 2% of the true error rates. When the errors are dependent, shadow regression is usually within 5% of the truth. Note that as the error rate increases, there is a tendency for shadow regression to underestimate the error rate. This is because at higher error rates, more reads have more than two errors, which are not captured because we only use shadows that differ from the read of interest by two bases. This trend is more pronounced in the dependent case as it is easier to have multiple errors in a read if the first error induces later ones. Figure 4 shows the position-specific error rate estimates given by shadow regression, which are very similar to estimates from mismatch counting, and track the true error rates very closely.

**Figure 5:**
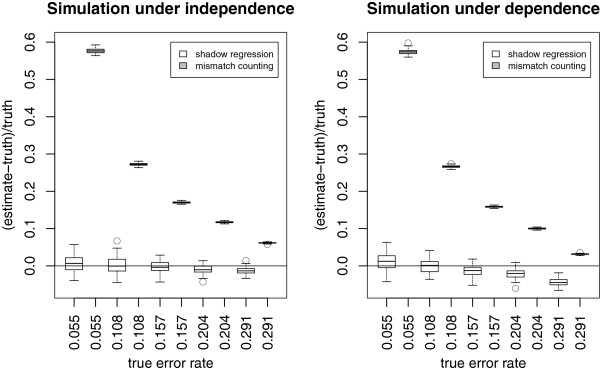
**Simulations: same as Figure **3**, but assumes that the true genome sequence of the sample of interest differs from the reference genome.** There is a single base pair difference per 1000 base pairs on average.

**Figure 6:**
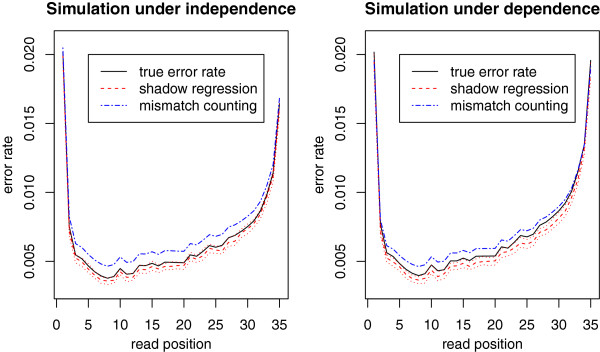
**Simulations: same as Figure **4**, but assumes that the true genome sequence of the sample of interest differs from the reference genome.** There is a single base pair difference per 1000 base pairs on average

The simulations presented this far assumed that the true genome sequence of the sample is exactly the same as that of the reference genome. In practice this is seldom the case. To assess the performance of shadow regression and mismatch counting when the sample genome sequence differs from the reference genome, we performed a simulation where we assumed that there is a single base pair difference between the sample genome and the reference genome every 1000 base pairs on average, a realistic value for human polymorphisms. This only affected the error rate estimates produced by mismatch counting since shadow regression does not use the reference genome. The results are shown in Figures 5 and 6. As expected, mismatch counting overestimates the error rate in this case.

### PhiX DNA data

**Figure 7:**
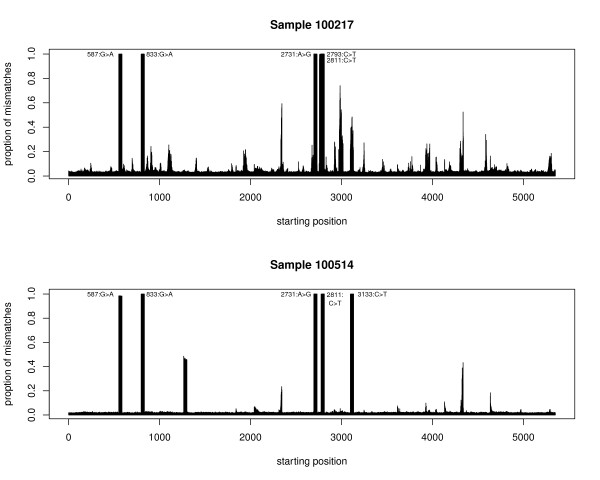
**Two PhiX samples run on Illumina Genome Analyzer II: Proportion of reads containing mismatches by starting position.** For a few positions, almost 100% of the reads covering them do not match the reference genome, substantially inflating the error rate estimates given by mismatch-counting. The widths of the bars reaching almost 1 (100%) and containing 1 mismatch are 36 bases, which is the read length for the PhiX samples.

Next, we evaluated our method using sequencing data of the bacteriophage PhiX 174RF1 (PhiX) DNA, whose complete sequence is known. DNA from PhiX is sometimes sequenced in one lane of Illumina flowcells to calibrate quality scores of the base caller [21] (Supplementary information page 7). We obtained Illumina data for two PhiX samples from the Center for Cancer Computational Biology at DFCI. Figure 2 shows that shadow regression fit reasonable lines to the PhiX samples and we would expect accurate error rate estimates from it. Mismatch counting first gave much higher error rate estimates than shadow regression. On further inspection, we realized that this was caused by several mutations in the PhiX genome. Because the coverages of PhiX samples were in the thousands, differences between the reference genome and the actual PhiX sequence in a few positions substantially inflated the error rates estimated by mismatch counting. This is shown clearly in Figure 7. At five places in each sample, almost all the reads differed from the reference genome. Closer examination of the data confirmed that at each of these places, almost all the mismatched reads had the same difference between the actual reads and the reference genome, leading us to conclude that the difference was due to real mutations rather than sequencing errors. Once we incorporated these mutations into the reference genome, mismatch counting and shadow regression gave error rate estimates that are much closer to each other (Table 1). The percentage of mismatched reads was much higher than the background at some other positions, although not nearly as high as 100%. These may be due to mixed PhiX species in the PhiX sample and other irregularities introduced in the sample processing steps, and explains the higher estimates given by mismatch counting compared to shadow regression. Our findings here demonstrate the advantage of shadow regression over methods that depend on the reference genome. Bullard and colleagues [10] found that PhiX calibration did not improve the detection of differentially expressed genes in mRNA-seq experiments and yielded fewer high quality reads per lane. Our analysis showed that this may be due to mutations in PhiX and that the reference sequence used in the calibration step can be different from the actual sequence of PhiX used. This is corroborated by the estimate given by [22], indicating that PhiX undergoes 1.0 × 10^−6 ^substitutions per base per round of copying.

**Table 1:** Estimated error rates (as a percent) in PhiX samples

**Sample ID**	**Shadow regression**	**mm**	**mm / new genome**
100217	2.613 (2.264, 2.962)	7.466	4.429
100514	1.416 (1.331, 1.500)	5.706	2.244

The intervals in parentheses for shadow regression estimates are the 95% confidence intervals of the estimates. The column “mm” gives the mismatch counting estimates using the standard PhiX reference genome. The column “mm / new genome” gives the mismatch counting estimates using the PhiX genome corrected for mutations shown in Figure 7.

### mRNA-seq: MAQC brain experiment 2 using auto calibration

**Figure 8:**
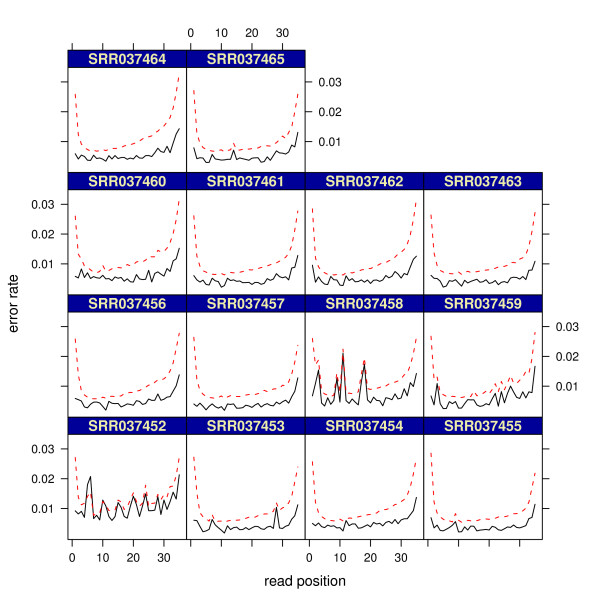
**MAQC brain experiment 2 using auto calibration: position-specific error rate estimates.** Dashed red lines are mismatch counting estimates. Solid black lines are shadow regression estimates.

We applied shadow regression to Illumina mRNA-seq data from the MAQC project [23]. A scatter plot of read and shadow counts for one of the samples is shown in Figure 1. Specifically, we used shadow regression to estimate the per-read and position-specific error rates for fourteen samples on two flow-cells (SRX016366 and SRX016368) run on the Illumina 1G Genome Analyzer, and results are shown in Table 2 and Figure 8 respectively. Most per-read error rates fell between 15% and 20%. For the two samples with higher than 20% per-read error rates, their corresponding position-specific error rates were not as smooth as other samples and they had substantial differences in neighboring positions. In all fourteen samples, mismatch counting gave higher error rate estimates than shadow regression, which is expected because mismatch counting classifies genuine differences between the sample of interest and the reference genome as errors.

**Table 2:** MAQC brain experiment 2 using auto calibration: per-read error rate estimates

**Sample**	**sr estimate**	**sr standard error**	**mm estimate**
SRR037452	0.27	0.0037	0.35
SRR037453	0.16	0.0031	0.25
SRR037454	0.16	0.0041	0.27
SRR037455	0.15	0.0027	0.23
SRR037456	0.17	0.0040	0.27
SRR037457	0.15	0.0031	0.24
SRR037458	0.24	0.0031	0.33
SRR037459	0.20	0.0036	0.28
SRR037460	0.20	0.0051	0.32
SRR037461	0.18	0.0041	0.27
SRR037462	0.19	0.0049	0.29
SRR037463	0.18	0.0041	0.27
SRR037464	0.19	0.0054	0.31
SRR037465	0.18	0.0038	0.26

Shadow regression is abbreviated “sr”. Mismatch-counting is abbreviated “mm”.

### mRNA-seq: Encode transcriptome

**Figure 9:**
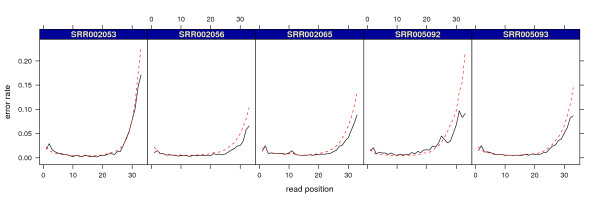
**Encode project mRNA-seq data: Position-specific error rate estimates.** Dashed red lines are mismatch counting estimates. Solid black lines are shadow regression estimates.

We applied shadow regression to a second Illumina mRNA-seq data set, this time five samples of human cell line K562 from the Encode project (SRX000570) [24], also run on the Illumina 1G Genome Analyzer. A scatter plot of read and shadow counts for one of the samples is shown in Figure 1. Results are shown in Table 3 and Figure 9. We found much higher error rates in these samples with per-read estimates around 40% to 50% compared to around 20% for the MAQC samples. The position-specific error rates were similar to MAQC samples in early cycles but increased dramatically after cycle 25, thus inflating the per-read error rates.

**Table 3:** Encode project mRNA-seq data: per-read error rate estimates

**Sample**	**sr estimate**	**sr standard error**	**mm estimate**
SRR002053	0.46	0.0065	0.63
SRR002056	0.33	0.0032	0.45
SRR002065	0.40	0.0041	0.55
SRR005092	0.52	0.0073	0.73
SRR005093	0.43	0.0046	0.56

Shadow regression is abbreviated “sr”. Mismatch-counting is abbreviated “mm”.

### Mutation screening: re-sequencing

**Figure 10:**
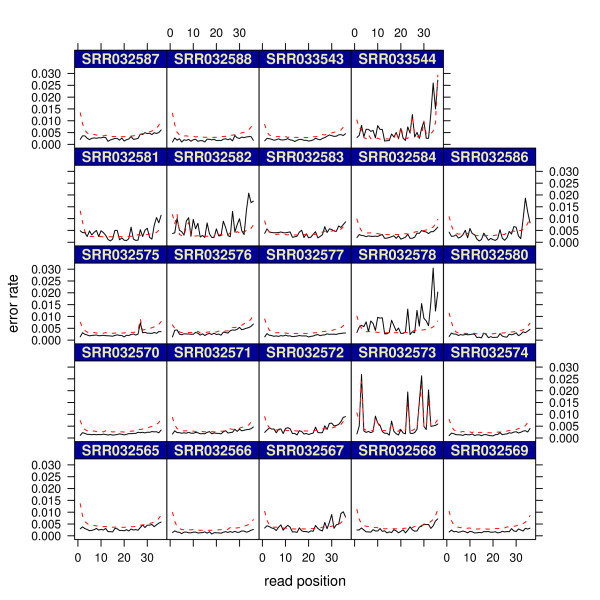
**Hu re-sequencing data.** Dashed red lines are mismatch counting estimates. Solid black lines are shadow regression estimates.

In addition to mRNA-seq, we also applied shadow regression to re-sequencing data from 24 samples as described in Hu *et al*. [25]. A novel multiplex PCR method and next generation sequencing were combined to screen patients with X-linked mental retardation for mutations in 86 genes. Sequencing was done using Illumina Genome Analyzer II. A scatter plot of read and shadow counts for one of the samples is shown in Figure 1. We used shadow regression to estimate the error rates in these samples, and results are shown in Figure 10 and Table 4. The error rates in this data set are in general lower than the two mRNA-seq data sets, possibly due to improvements in the Genome Analyzer II. Shadow regression gave lower per-read estimates than mismatch counting for most samples as seen in the two mRNA-seq data sets. In about half the samples, shadow regression estimated much higher error rates in the last few cycles than mismatch counting.

**Table 4:** Hu re-sequencing data: per-read error rate estimates

**Sample**	**sr estimate**	**sr standard error**	**mm estimate**
SRR032565	0.08	0.0004	0.15
SRR032566	0.06	0.0002	0.11
SRR032567	0.08	0.0003	0.12
SRR032568	0.07	0.0006	0.13
SRR032569	0.06	0.0003	0.12
SRR032570	0.07	0.0011	0.11
SRR032571	0.07	0.0003	0.12
SRR032572	0.08	0.0003	0.12
SRR032573	0.16	0.0005	0.19
SRR032574	0.06	0.0007	0.10
SRR032575	0.09	0.0007	0.13
SRR032576	0.11	0.0006	0.14
SRR032577	0.08	0.0007	0.12
SRR032578	0.19	0.0004	0.13
SRR032580	0.07	0.0004	0.11
SRR032581	0.11	0.0002	0.11
SRR032582	0.15	0.0002	0.13
SRR032583	0.11	0.0003	0.13
SRR032584	0.08	0.0004	0.14
SRR032586	0.08	0.0001	0.13
SRR032587	0.09	0.0005	0.14
SRR032588	0.07	0.0003	0.13
SRR033543	0.08	0.0005	0.13
SRR033544	0.15	0.0002	0.16

Shadow regression is abbreviated “sr”. Mismatch-counting is abbreviated “mm”.

### Application to Serial Analysis of Gene Expression (SAGE)

SAGE is a powerful technique for the examination of genome-wide expression levels that involves considerable sequencing of concatenated ten base pair long tags corresponding to transcripts [26]. Velculescu and colleagues [27,28] estimated the magnitude of sequencing errors in a SAGE library using error estimates obtained from studies in yeast. Previous work by the same group compared the 60,633 transcripts in the SAGE yeast library to the known yeast genome, and determined that 56,291 tags match the tags predicted by the sequence of the yeast genome, 88 of the tags match the mitochondrial genome, and an additional 91 tags match a plasmid present in yeast [27]. The remaining 4,163 tags did not match the yeast genome and were therefore considered to be the result of sequencing errors. As these 4,163 tags represent 6.8% of the total library, Velculescu reports that 6.8% of all tags are thought to have a sequencing error. This estimate may be conservative because it only considers novel tags introduced by sequencing errors. Sequencing errors which create additional copies of tags already present in the library rather than novel tags have not been considered in this calculation of the error rate. Additionally, this error rate may include legitimate tags not matching the yeast genome databases because of single nucleotide differences between the yeast strain analyzed by SAGE and those used for genome sequencing, or because of incomplete genome sequencing. We applied shadow regression to sixteen human samples of pancreatic and colorectal tissue available from the NCBI SAGEMap website. A scatter plot of read and shadow counts for one of the samples is shown in Figure 1. Estimated error rates are shown in Table 5. These error rates are slightly higher than the reported 6.8%, as expected, confirming the accuracy of the shadow regression method. These error rates are lower than in previous data sets as conventional sequencing was used in these data sets instead of next generation sequencing.

**Table 5:** SAGE data: human colorectal and pancreatic samples

**Sample**	**sr estimate**	**sr standard error**
91-16113	0.04	0.0064
96-6252	0.02	0.0053
ASPC	0.04	0.0041
CACO2	0.09	0.0094
CAPAN1	0.08	0.0117
H126	0.09	0.0082
HCT116	0.09	0.0111
HX	0.09	0.0085
NC1	0.08	0.0064
NC2	0.07	0.0052
PANC1	0.08	0.0063
PL45	0.07	0.0062
RKO	0.10	0.0104
SW837	0.07	0.0081
TU102	0.07	0.0071
TU98	0.08	0.0087

Per-read error rate estimates and standard errors from shadow regression.

## Conclusions

We established a simple, general, flexible and robust methodology to estimate error rates for any of the existing second-generation sequencing technologies producing short read sequences. The fundamental advance behind the proposed methodology is our observation that there is a linear relationship between the frequency of short reads and the frequencies of their ‘neighboring’ reads, where neighbors are defined by sequence similarity. This linear increase reflects sequencing errors, as frequently occurring tags cast larger “shadow” of errors on neighboring tags. This observation holds true of SAGE libraries and seems to hold universally across second-generation sequencing platforms with moderate or high sequencing depths as well. Because shadow regression estimates the slope robustly, the error rate estimates are not influenced by shadows that are error-free.

The shadow regression method does not require mapping reads to a reference genome. This has significant computational advantages, but more importantly it can address critical biological needs. For example, the ability to measure error rates independent of a reference genome can be critical in experiments designed to detect unknown species, as in threat detection, and in experiments investigating many species simultaneously, as when studying the microbiome. When the reference genome is prone to errors, for example because of a relatively high mutation rate of the system studied, we showed that the method of using the differences between the sample of interest and the reference genome as proxy for sequencing errors can produce estimates that are substantially inflated, while shadow regression is not affected by such differences.

We also showed the accuracy of shadow regression through simulation studies and analyses of the PhiX and SAGE data. We applied shadow regression to mRNA-seq, DNA sequencing, mutation screening and SAGE, and demonstrated that this approach can be immediately used to evaluate sequencing error rates in different applications as they are generated. Even though our next-generation sequencing examples are all from the Illumina platform, shadow regression can be applied to other sequencing platforms as well.

We hope that the availability of a simple and computationally effective way of computing error rates at the level of single sequencing experiments will contribute significantly to quality control, proper analysis and experimental design of second-generation sequencing efforts.

## Competing interests

The authors declare that they have no competing interests.

## Authors’ contributions

XVW co-developed the statistical methods, wrote the ShadowRegression R package, performed data analysis, and drafted the manuscript. NB co-developed the statistical methods and edited the manuscript. JD co-developed the statistical methods. RS designed the PhiX experiment and edited the manuscript. GP co-developed the statistical methods, edited the manuscript, and coordinated the research. All authors read and approved the final manuscript.
